# Femoral or Axillary Cannulation for Extracorporeal Circulation during Minimally Invasive Heart Valve Surgery (FAMI): Protocol for a Multi-Center Prospective Randomized Trial

**DOI:** 10.3390/jcm12165344

**Published:** 2023-08-17

**Authors:** Jacqueline Kruse, Miriam Silaschi, Markus Velten, Maria Wittmann, Eissa Alaj, Ali El-Sayed Ahmad, Sebastian Zimmer, Michael A. Borger, Farhad Bakhtiary

**Affiliations:** 1 Department of Cardiac Surgery, University Hospital Bonn, 53127 Bonn, Germany; miriam.silaschi@ukbonn.de (M.S.);; 2 Department of Anesthesiology and Intensive Care Medicine, University Hospital Bonn, 53127 Bonn, Germany; 3 Department of Cardiology, University Hospital Bonn, 53127 Bonn, Germany; 4 Department of Cardiac Surgery, Leipzig Heart Center, 04289 Leipzig, Germany

**Keywords:** FAMI, femoral cannulation, axillary cannulation, perfusion strategies, extracorporeal circulation, minimally invasive surgery

## Abstract

Background: Minimally invasive heart valve surgery via anterolateral mini-thoracotomy with full endoscopic 3D visualization (MIS) has become the standard treatment of patients with valvular heart disease and low operative risk over the past two decades. It requires extracorporeal circulation and cardioplegic arrest. The most established form of arterial cannulation for MIS is through the femoral artery and is used by most surgeons, but it is suspected to increase the risk of stroke through retrograde blood flow. An alternative route of cannulation is the axillary artery, producing antegrade blood flow during extracorporeal circulation. Methods: Femoral or axillary cannulation for extracorporeal circulation during minimally invasive heart valve surgery (FAMI) is a multicenter randomized controlled trial designed to determine whether axillary cannulation is superior to femoral cannulation for the outcome of a manifest stroke within 7 days postoperatively. The target sample size was 848 participants. Patients ≥ 18 years of age, with valvular regurgitation or stenosis scheduled for minimally invasive surgery via anterolateral mini-thoracotomy, were randomized to axillary cannulation (treatment group) or to femoral cannulation (standard care). Patients were followed up for seven days postoperatively. A CT scan was performed pre-operatively to screen patients for vascular calcifications and to assess the safety of femoral cannulation. The standard of care is femoral artery cannulation, but is performed only in patients without significant vascular calcifications or severe kinking of the iliac arteries and in patients with sufficient vessel diameter. The cannulation is performed via Seldinger’s technique, and the vessel closed percutaneously using a plug-based vascular closure device. Only patients without significant vascular calcifications are considered for femoral cannulation, as an increased risk of stroke is assumed. In patients with vascular calcifications, axillary cannulation is the standard of care to avoid these risks. Retrospective studies have hinted that, even in patients without vascular calcifications, there may be a lower stroke risk with axillary cannulation compared to femoral cannulation. We present a protocol for a multi-center randomized trial to investigate this hypothesis. Discussion: To date, evidence on the best access for peripheral artery cannulation during minimally invasive heart valve surgery has been scarce. Patients may benefit from axillary cannulation for extracorporeal circulation in terms of stroke risk and other neurological and vascular complications, though femoral cannulation is the gold standard. The aim of this study is to determine the risks of peri-operative stroke in a prospective randomized comparison of femoral vs. axillary cannulation.

## 1. Introduction

### 1.1. Background

Minimally invasive heart valve surgery via anterolateral mini-thoracotomy with full endoscopic 3D visualization (MIS) has become the standard treatment for patients with valvular heart disease and low operative risk in experienced centers in Europe over the past two decades. Compared to conventional median sternotomy, this surgical technique has been shown to have a low postoperative complication rate in terms of postoperative pain, length of ICU stay (including length of postoperative ventilation), and overall length of hospital stay [1,2].

In minimally invasive heart valve surgery, the heart–lung machine for extracorporeal circulation is usually not connected (hence “cannulated”) centrally to the ascending aorta, but via peripheral vessels. Extracorporeal circulation is needed for the induction of cardiac arrest and opening the cardiac chambers to enable valve replacement or valve reconstruction.

**Figure 1:**
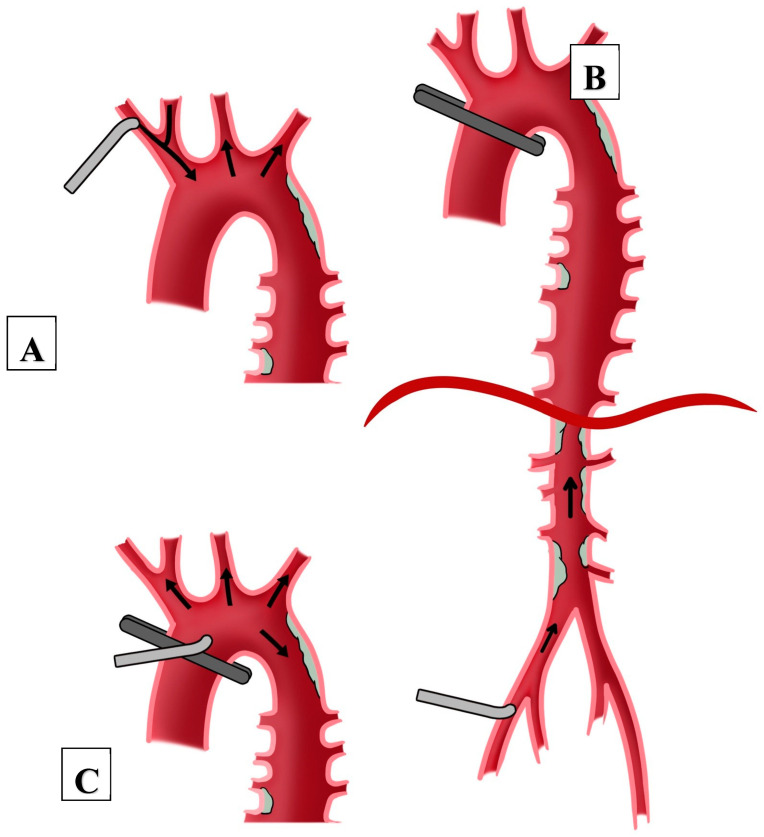
(**A**) schematic illustration of peripheral cannulation of subclavian artery. (**B**) Femoral artery with retrograde perfusion; (**C**) central cannulation with antegrade perfusion. The arrows in this figure indicate the direction of blood flow.

The most established form of cannulation for MIS is via the femoral artery. This is considered a safe cannulation route in patients without significant vascular calcifications, which can be seen in routine pre-operative computed tomography (CT) scans. An alternative cannulation route is through the axillary artery. In order to use this access, an exposure of the vessel is required, which is more time-consuming compared to femoral cannulation and most often requires an additional small surgical incision below the clavicular bone, as Seldinger’s technique is less preferred. Retrospective studies suggest an increased stroke risk with femoral cannulation, even in patients without significant vascular calcifications [3,4]. This is explained by the concept of “retrograde” and “antegrade” perfusion in the aorta during cardiopulmonary bypass and cardioplegic arrest (see Figure 1), which provides a possibility of mobilizing calcium and debris upward to the supraaortic vessels. Significant kinking or stenosis may also lead to hypoperfusion proximally or may carry a risk of vessel injury during cannulation. 

### 1.2. Objectives

The aim of this study was to determine the risks of peri-operative stroke in a prospective randomized comparison of femoral vs. axillary cannulation in minimally invasive heart valve surgery.

### 1.3. Trial Design

This trial is planned as a multi-center, randomized, prospective, controlled trial to achieve data of the highest scientific quality. The study data will be collected and managed using REDCap electronic data capture tools hosted at Vanderbilt University [5,6]. A blinding for the investigator and patient is evidently not possible. Mock-incisions are considered as unethical; thus single-blinding is also not possible. Stroke and disability are endpoints which will be assessed through objective measures (CT scans, NIH Stroke Scale, Modified Rankin scale). The trial will be performed following all legal requirements and scientific standards, especially the Good Clinical Practice (GCP) guideline, including onsite and remote monitoring of the data, adequate training of the staff, standardized safety management, and quality management of the trial.

### 1.4. Methods

#### Study Settings Pre-Operatively

Patients with heart valve disease (aortic, and/or tricuspid, and/or mitral) with indication for surgery will receive a CT-scan, performed pre-operatively to prepare patients and assess the safety of femoral and axillary cannulation. A recent meta-analysis suggested a reduced risk of stroke in minimally invasive heart valve surgery using pre-operative CT scans [7].

**Figure 2:**
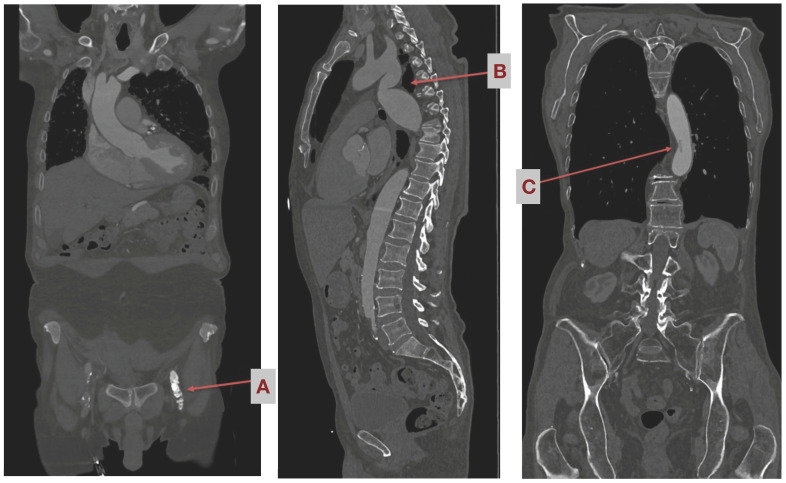
Incidental finding with contra-indications for femoral cannulation and retrograde blood flow as seen in preoperative CT angiography scan. (**A**) Occlusion of common femoral artery, (**B**) Stenosis of aortic isthmus, (**C**) Thrombus in the abdominal aorta.

For screening purposes, a special CT angiography is carried out from neck to groin. It is carried out using a contrast agent, and imaging is ECG-triggered. In addition, a reconstruction is shown in a coronary sectional plane (see Figure 2). CT screening is routinely applied in all MIS patients and may show accidental findings that are contraindicative to femoral cannulation. In cases of severe renal impairment, the need for a contrast agent may be waived; however, it is preferable to obtain CT images using a contrast agent. 

### 1.5. Eligibility Criteria

Patients with valvular insufficiency or stenosis who are planned for minimally invasive surgery via anterolateral mini-thoracotomy, as well as patients who are suitable for both femoral and axillary cannulation using the heart–lung machine according to their CT scan (i.e., no relevant calcifications of the pelvic leg vessels and the aorta, sufficient vessel diameter (see Figure 2)) are eligible for enrolment. 

Patients with vascular access sites that are anatomically unsuitable for femoral or axillary artery cannulation, vascular calcifications, or higher-grade stenoses in the supra-aortic vessels, or patients with pre-interventional complications at the vascular access site (before the actual minimally invasive valve replacement/reconstruction procedure), are not eligible. 

Inclusion criteria: Patients with valvular regurgitation or stenosis scheduled for minimally invasive surgery via anterolateral mini-thoracotomy. Patients suitable for both femoral and axillary cannulation of the heart–lung machine (i.e., no relevant calcifications of the iliac leg vessels and the aorta, sufficient vascular diameter). Consent of the patient after detailed explanation of the study. Age of patient: >18 years old.

Exclusion criteria: A vascular access site that is anatomically unsuitable for cannulation in the femoral or axillary artery. Patients with vascular calcifications, higher-grade stenoses in the para-aortic vessels, pre-interventional complications at the vascular access site (before the actual minimally invasive valve replacement/reconstruction procedure), unstable active bleeding, bleeding diathesis, or significant unmanageable anemia also inhibit the capacity of the patient to give consent. 

### 1.6. Surgical Procedure Step-by-Step

At the beginning of the procedure, the patient is fully heparinized (400 I.E. Heparine/kg body weight). Successful heparinization is measured using “Activated clotting time”, which should exceed 450 s and be monitored repeatedly. Extracorporeal circulation (ECC) is established percutaneously via femoral vessels under transesophageal echocardiography (TEE) guidance. Alternatively, cannulation of the axillary artery after cut-down in the infraclavicular groove or under ultrasound guidance via Seldinger’s technique can be performed. The arterial cannula diameter ranges from 16 to 20 Fr, and is needed for the generation of enough flow through extracorporeal circulation at outlet pressures below 300 mmHg. 

We perform the procedure in normothermia; however, if need be, mild hypothermia may be applied. Venous cannulation always includes femoral access in cases of >80 kgs of body weight or tricuspid valve surgery, and an additional venous neck cannula may be placed through the internal jugular vein. 

Then, a right thoracotomy is performed through 2nd or 3rd intercostal space over approximately 5 cm. Access through the ribs is needed during apnea, as well as disconnection of the endotracheal tube. A soft tissue retractor is used and a 3D optic is inserted through a port one intercostal space above the thoracotomy incision. Continuous inflation of CO_2_ via the endoscopy port is applied. The pericardium is opened above the phrenic nerve, and stay sutures are placed for visualization. 

Separation of the aorta from the pulmonary artery, release of the adhesions on the aorta, and insertion of the transthoracic aortic clamp via another stab incision craniolateral from the surgical incision are performed. Vents are placed on the ascending aorta and, in cases of aortic valve surgery, the right upper pulmonary vein. Cross-clamping of the aorta and administration of 1500–2000 mL of crystalloid cardioplegia solution via the aortic root are carried out. If aortic valve (AV) regurgitation is severe, cardioplegia may be administered directly into the ostia.

**Figure 3:**
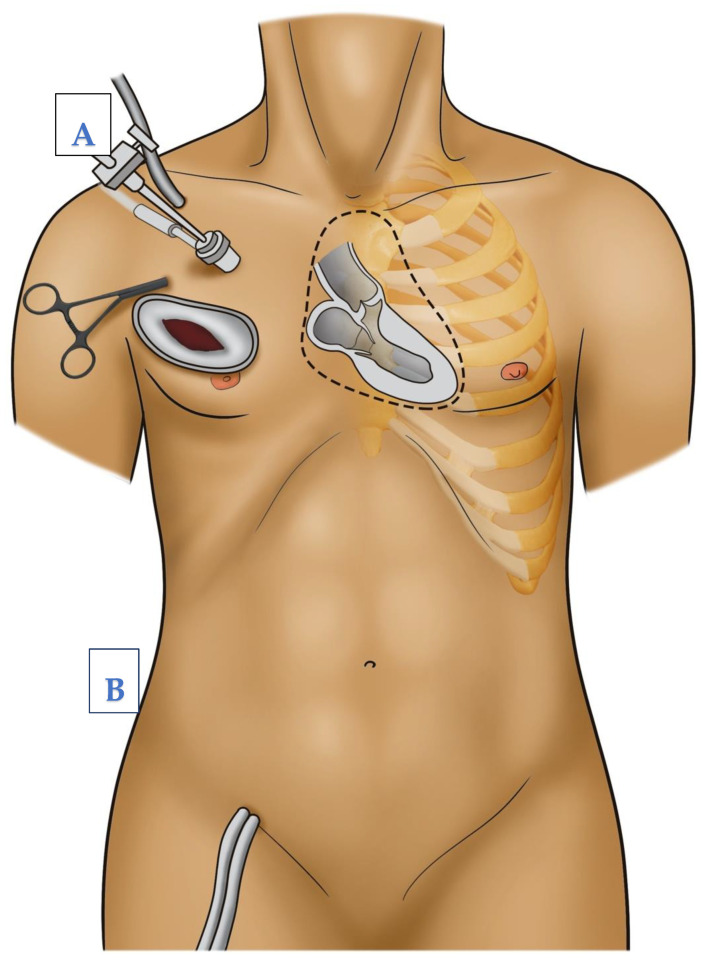
Operative setup and possible cannulation sites for minimally invasive heart surgery via right anterior thoracotomy. (**A**) Axillary cannulation, (**B**) femoral cannulation.

- In cases of aortic valve surgery, after the induction of sufficient cardioplegia, the aorta is incised transversally. Then, the aortic valve procedure, repair, or replacement is performed as needed. Afterwards, control of the coronary ostia and control of the leaflets, then double row closure of the aorta, are performed
- In cases of mitral valve surgery, a retraction suture is placed at the level of the right atrium to expose the interatrial grove. The left atrium is then opened at the level of the interatrial grove and exposed using a retractor, which is stabilized by another stab incision, parasternal at the level of the same intercostal space used for thoracotomy (see Figure 3). Then, the mitral valve procedure, repair, or replacement is performed as needed. Afterwards, the waterprobe of the left atrium is closed using continuous double layered sutures.

Placement of a temporary pacemaker electrode to the right ventricle is necessary before releasing the aortic cross clamp. Next, a de-airing maneuver is performed (recruitment of ventilation and temporarily reducing venous drainage with strong suction on the left ventricular vent as well as at the aortic vent). The aortic cross-clamp is released. 

After sufficient reperfusion, ventilation is initiated and establishment of a stable rhythm occurs, then extracorporeal circulation is weaned gradually and terminated. The venous line may be decannulated using one deep skin suture and manual compression of the vessel. The arterial femoral line is decannulated using the MANTA percutaneous vascular closure system (Teleflex, Morrisville, NC, USA).

In cases of axillary cannulation, the arterial line is removed and previously-placed purse string sutures are tied down. The vessel is inspected for any complications (stenosis dissection, etc.), and afterward, the axillary wound may be closed (3–4 cm). Heparin is antagonized using Protamine. The surgical incision, pleura, and pericardium are inspected for any signs of bleeding.

Two chest drains are routinely placed through the same incisions used for the endoscope and the aortic clamp, one pleural drain and one pericardial drain. The ribs are adapted using a thick suture to prevent lung hernia. After that, the wound is closed in a layered fashion. Incisions may be infiltrated with local anesthetics and the patient may be extubated on the operating table directly afterwards, which is routine at out center. Figure 1 shows the operative setup of MIS with surgical incisions and possible cannulation sites.

## 2. Outcomes 

### 2.1. Primary Endpoint

Manifest stroke (confirmed by imaging or non-transient new neurological deficit) can occur within 7 days postoperatively.

### 2.2. Secondary Endpoints

- Rate of transient ischemic attacks;
- Modified Rankin Scale (0–6 points) at discharge;
- Rate of postoperative delirium (mean CAM-ICU score);
- Neurological status assessed by mean NIH Stroke Scale at discharge;
- Rate of seizures generalized and focal;
- Hours on ventilator post-procedure;
- Long-term survival rate (1 year);
- Rate of access-site or access-related bleeding;
- Rate of wound healing disorders in the cannulation area;
- Rate of iatrogenic aortic dissection;
- Thromboembolic events of limbs;
- Rate of pseudoaneurysm or other vascular complications at access site.

## 3. Study Visits

The study will be conducted at high-volume heart valve centers specializing in MIS with >250 minimally invasive heart valve procedures performed annually per center. All patients admitted for minimally-invasive valve surgery at the participating centers will be screened and assessed for eligibility. CT scans will be performed as per the standard of care, and consent will be obtained after the CT scans show eligibility for both cannulation routes. The course of study is listed in Table 1. The modified Rankin scale (mrs) and NIH stroke scale are routinely assessed as per the protocol outlined in Table 1. The mrs measures the degree of disability after a stroke, and the NIH stroke scale quantifies the stroke’s severity. The members of the study team performing these assessments must obtain a training certificate prior to performing assessments on the study patients. 

### 3.1. Provisions for Post-Trial Care

There will be no compensation for patients since both procedures are well established and there is no off-label-use. No additional insurance for participating patients is needed as the cannulas for the heart–lung machine are used within the scope of their instructions for use, and the cannulation strategy in itself is not experimental.

### 3.2. Sample Size and Statistical Analysis

The assumed stroke rate in the intervention group is 1%, and in the control group 4%, based on reported outcomes from a propensity-matched retrospective study of both techniques consisting of >1500 patients [3,4] (Murzi et al. [3,4]. Based on our calculations, 424 patients per group must be included (i.e., 848 patients in total) to test the primary endpoint with a power of 80%. Overall, we assumed a slightly lower stroke rate in the control group than previously reported (4% instead of 5%) to ensure enrolment of enough patients to achieve sufficient power to test the primary endpoint if the stroke rate turns out to be lower. 

The efficacy of the primary endpoint will be calculated using the chi-square test. The endpoint will be measured and tested using the stroke rate up to 7 days postoperatively in both groups. All other categorical endpoints will be measured using chi-square tests. Secondary endpoints with continuous variables will be compared using the t-test for comparison of means, Kaplan–Meier for survival (log-rank) and chi-square for categorical endpoints. A combined endpoint may be created, consisting of stroke, delirium, and access-site-related complications at 30 days to further assess overall safety of the cannulation routes and to achieve a higher level of sensitivity in detecting complications by both treatments.

An interim analysis will be performed after inclusion of 50% of the patients (424). If a significantly higher stroke rate is shown in one group which exceeds the expected stroke rate, the trial may be stopped and concluded for safety reasons and obvious superiority of one technique. If the actual stroke rate differs from the assumed stroke rate, sample size calculation may be repeated on the basis of interim analysis, and enrolment will be adjusted accordingly.

**Figure 4:**
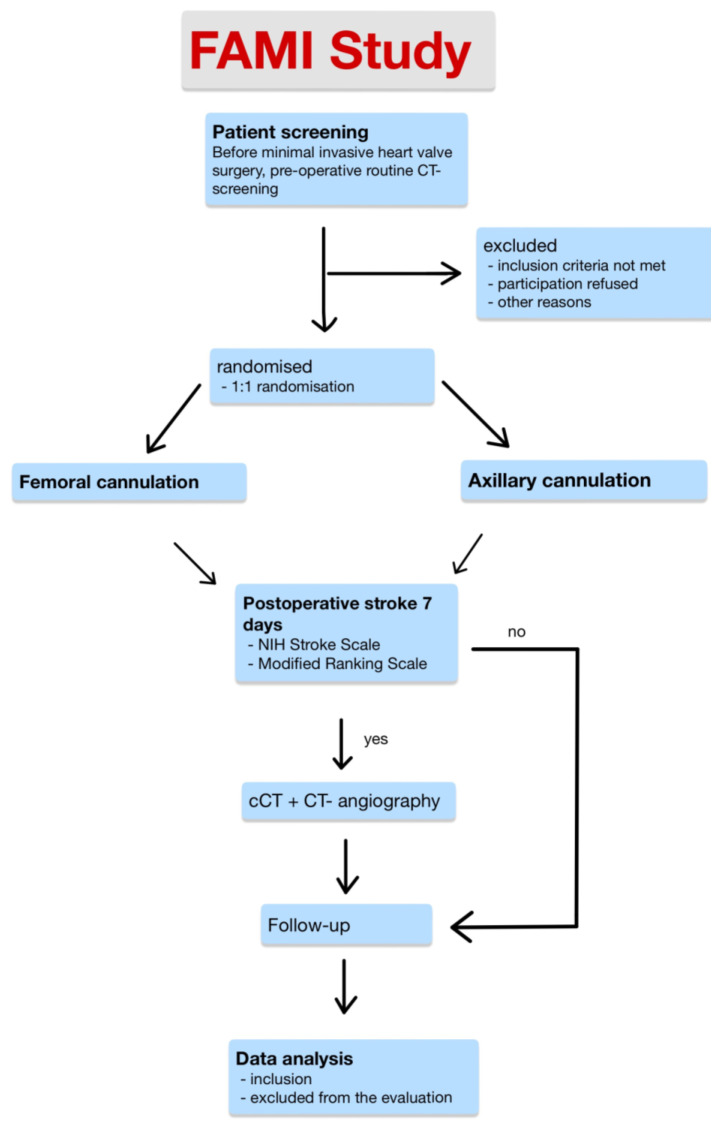
FAMI study flow-chart detailing the screening, enrolment, and study process.

A study flow-chart detailing the screening, enrolment, and study process can be seen in Figure 4.

## 4. Data Management

In this study, the randomization of patients assigned to different interventions was conducted using an online randomization tool. The use of such a tool ensures a fair and unbiased allocation of participants to the intervention groups. The randomization process involved assigning each participant a unique identifier and then using the online tool to generate a random sequence of assignments. This method helps to minimize selection bias and ensures that each participant has an equal chance of being assigned to any of the intervention groups. The online randomization tool used in this study provides a transparent and efficient way to allocate participants, enhancing the validity and reliability of the research findings. By employing this randomization method, we aim to ensure the integrity and rigor of our study design.

A simple randomization process was performed using the REDCap database in this study. The purpose of the randomization was to assign participants to different treatment groups in a completely unbiased manner. Therefore, we used the simple randomization application. This method ensures that each participant has an equal chance of being assigned to any of the treatment groups, minimizing potential biases and increasing the reliability of the study results. The randomization process was conducted using the REDCap database, which provides a secure and efficient platform for data management in research studies.

The data will be stored on a secure server in the protected environment of the Study Centre Bonn. A full-time data manager is already employed by our department and is familiar with data export to REDCap. Data handling will be conducted in accordance with the GCP guidelines and current European data legislation. A GCP-compliant eCRF with secured access and an implemented audit trial will be used. Central monitoring with plausibility checks, as well as on-site monitoring, will be implemented following a risk-adapted approach to ensure high data quality. After clearance and final analysis of the data, as well as the publication of study results, the data will be made publicly available.

## 5. Plans to Promote Participant Retention and Complete Follow-Up

After hospital discharge post-surgery, follow-up will be telephone-based; therefore, adherence to follow-up is expected to be high. If the access route of cannulation is switched to another route, the mode of follow-up and clinical testing will not differ from the rest of the study population. There will be intention-to-treat analysis and per-protocol analysis at the time of publication to account for changes in the mode of cannulation.

## 6. Feasibility of Recruitment/Trial Sites

The study will be conducted at high-volume heart valve centers specializing in minimally invasive valve surgery. All patients admitted for minimally-invasive valve surgery at the participating centers will be screened and assessed for their eligibility. CT scans are performed as per the standard of care, and consent will be obtained after CT scans show eligibility for both cannulation routes.

## 7. Discussion

There is evidence that, even in patients without atherosclerotic vascular changes, axillary cannulation may be more beneficial with regard to the development of peri-operative strokes during MIS using extracorporeal circulation. So far, these have been retrospective observations. 

Murzi et al. (2013) conducted a study with 1280 patients comparing antegrade and retrograde flow in minimally invasive mitral valve replacement over a period of 9 years. Retrograde perfusion was shown to increase the risk of stroke (5 vs. 1%) and postoperative delirium (14 vs. 5%). However, the results should be interpreted carefully, since in one-third of patients who underwent retrograde cannulation, the aorta was occluded for cardiac arrest via a balloon occlusion and not via a vascular clamp [3]. 

In another study by Murzi et al. (2016), 1632 patients who had undergone minimally invasive mitral valve replacement were retrospectively reviewed. Anterograde and retrograde cannulation were examined with regard to the postoperative outcome. Patients under 70 years of age were not found to have a significantly higher risk of neurological complications with retrograde perfusion. The patients who were over 70 years old all had increased atherosclerotic risk factors and had a higher risk of stroke [4]. Grossi EA et al. (2012) also showed that patients with severe peripheral vascular disease have a higher risk of stroke as a result of retrograde arterial perfusion. With the decreasing usage of retrograde cannulation, the total frequency of postoperative neurological episodes decreased significantly over the course of the study (from 4.7% to 1.2% over 12 years) [8].

To date, there is only a small number of retrospective studies on the topic of femoral (retrograde) vs. axillary (antegrade) cannulation. Especially in patients without significant atherosclerotic vascular changes, it is not clear whether neurological complications can be avoided by axillary cannulation. Conducting a randomized study in patients with significant atherosclerotic burden would be considered unethical, since an increased stroke risk must be assumed. The studies described above indicated an increased risk with retrograde perfusion, but due to their retrospective nature and missing pre-operative CT data, these studies can only have limited significance. Also, only patients with mitral valve replacement were compared. We intend to analyze these outcomes for all minimally-invasive heart valve surgeries performed via mini-thoracotomy. In conclusion, there is a significant gap in the evidence with regard to cannulation strategies in minimally invasive heart valve surgery. Since more than 30,000 heart valve surgeries are performed annually in Germany, with a significant share being minimally invasive (59% in mitral valve surgery is currently minimally-invasive), reducing the risks of peri-operative stroke further seems to be an important contemporary task. The results of this study will influence guideline recommendations on perfusion strategy in MIS and cardiac surgery overall.

## 8. Trial Status

The trial is registered in the German clinical trials system (DRKS-ID: DRKS00030486) and was approved by the local ethics committee on 19 December 2022. The trial is currently enrolling patients.

## Figures and Tables

**Table 1:** Course of FAMI study.

	Course of Study			
	Inclusion	Reference	Visits	
Time	-t1 (Pre-OP)	t0 (Intra-OP)	t1–3: Post-OP	t7 or Date of Discharge: Follow Up
**Screening**	X	X		
**Informed consent**	X			
**Clinical examination**	X	X	X	X
**CT-angiography**	X		X *	X *
**Modified Rankin Scale**	X		X	X
**NIH Stroke scale**	X		X	X
**CAM-ICU**			X	X

* Cranial CT is only performed postoperatively if the patient presents with neurological symptoms.

## Data Availability

Not applicable.

## Abbreviations

MIS	minimal invasive surgery
CT	computer tomography
NIH Stroke Scale	National Institutes of Health Stroke Scale
CAM ICU Score	confusion assessment method for intensive care unit
FAMI	Femoral or axillary cannulation for extracorporeal circulation during minimally invasive heart valve surgery
